# Is Sham Laser a Valid Control for Acupuncture Trials?

**DOI:** 10.1093/ecam/neq009

**Published:** 2011-03-10

**Authors:** Dominik Irnich, Norbert Salih, Martin Offenbächer, Johannes Fleckenstein

**Affiliations:** ^1^Multidisciplinary Pain Center, Department of Anaesthesiology, University of Munich, Pettenkoferstraße 8A, 80336 Munich, Germany; ^2^Department of Pediatrics, Staedtisches Klinikum City of Munich/Harlaching, Germany; ^3^Physical Medicine, Rehabilitation and Prevention, Lindwurmstr, Munich, Germany

## Abstract

Methodological problems of acupuncture trials focus on adequate placebo controls. In this trial we evaluated the use of sham laser acupuncture as a control procedure. Thirty-four healthy volunteers received verum laser (invisible infrared laser emission and red light, 45 s and 1 J per point) and sham laser (red light) treatment at three acupuncture points (LI4, LU7 and LR3) in a randomized, double-blinded, cross-over design. The main outcome measure was the ratio of correct to incorrect ratings of treatment immediately after each session. The secondary outcome measure was the occurrence of deqi-like sensations at the acupuncture points and their intensity on a 10-fold visual analog scale (VAS; 10 being the strongest sensible sensation). We pooled the results of three former trials to evaluate the credibility of sham laser acupuncture when compared to needle acupuncture. Fifteen out of 34 (44%) healthy volunteers (age: 28 ± 10.7 years) identified the used laser device after the first session and 14 (41%) after the second session. Hence, both treatments were undistinguishable (*P* = .26). Deqi-like sensations occurred in 46% of active laser (2.34 VAS) and in 49.0% of sham laser beams (2.49 VAS). The credibility of sham laser was not different from needle acupuncture. Sham laser acupuncture can serve as a valid placebo control in laser acupuncture studies. Due to similar credibility and the lack of sensory input on the peripheral nervous system, sham laser acupuncture can also serve as a sham control for acupuncture trials, in order to evaluate needling effects *per se*.

## 1. Introduction

Laser acupuncture is defined as the stimulation of traditional acupuncture points with low-intensity, non-thermal laser irradiation. Although the therapeutic use of laser acupuncture is rapidly gaining popularity, objective evaluation of its efficacy in published studies is difficult because treatment parameters, such as wavelength, irradiance and beam profile, are seldom fully described [1]. Evidence for laser acupuncture-mediated effects comes from functional magnetic resonance imaging studies. Cho et al. demonstrated visual cortex activation in response to laser irradiation of acupuncture point BL-67 [2]. In contrast, no such response was found when the laser was placed in contact with the skin at the acupuncture point when not switched on. Even though similar intriguing correlations between activation of specific brain regions and needle stimulation have been demonstrated [3], such results do not prove efficacy. These results could not be reproduced in further trials using appropriate imaging techniques [4], thus leading to the retraction of Cho's publication [5], and so scepticism regarding the concept of low-intensity laser acupuncture therapy significantly increased [6, 7].

Laser acupuncture is thought to provoke nerve fiber activation; laser pulses of short duration were used in the past to assess the function of central and peripheral components of the nociceptive system, showing that laser-evoked potentials could be mediated through A*δ*- and C-fiber activation [8], which is also believed in needle acupuncture. The results of studies examining the effect of laser irradiation on such peripheral nerve fibers remain contradictory [1].

To evaluate clinical effects, sham laser acupuncture, a deactivated laser device, has been used as a placebo control in clinical laser acupuncture trials. The results of these trials have been contradictory, with some patients treated with sham laser experiencing remarkable clinical improvements [9]. Following the suggestions of a recent review, methodological limitations become evident [9]. The fact remains that sham laser acupuncture has never been validated when it comes to blinding properties, deqi sensation or credibility as main outcome measures. Therefore, this was our motivation to perform what we consider an overdue trial comparing sham laser acupuncture to laser acupuncture from a methodological perspective.

Sham laser acupuncture has also been used as a control for needle acupuncture trials [10–12]. The methodology of acupuncture trials raises several difficult issues, most notably the choice of appropriate control procedures [13–15]. Several different acupuncture controls have been proposed, including superficial needling, needling at inappropriate points or at non-acupuncture points, needling with blunt needles, off point and sham electro-stimulation and sham transcutaneous electrical nerve stimulation (TENS) [16]. However, recent acupuncture trials could not repeatedly demonstrate the superiority of needle acupuncture when compared to these control procedures [17–20]. One possible explanation for these results is the observation that the control treatments were not able to distinguish between specific effects from needle acupuncture (comprising mode or site specificity) and non-specific effects, that is, psychological and other physiological effects. Psychological effects might be due to cognitive and conditioning mechanisms, for example, caused by the time the practitioner spends performing the procedure or by the particularity of the needling ritual. Non-specific physiological effects are supposed to be caused by the physical, manual, invasive or tactile nature of the procedure; all of these potentially activating neurophysiological mechanisms [21–23]. For controls to be valid it is therefore necessary to avoid non-specific physiological activation in order to differentiate needle effects from non-specific effects.

Bringing these concepts together, we conducted this trial to determine if sham laser acupuncture could be distinguished from laser acupuncture and whether its credibility is equal to needle acupuncture based on an analysis of three recently performed trials.

## 2. Methods

### 2.1. Study Design

We conducted a randomized placebo-controlled, double-blinded cross-over trial at the Interdisciplinary Pain Center, Department for Anaesthesia, University of Munich, Germany, comparing the validity of a sham laser with actual laser acupuncture treatment.

**Figure 1:**
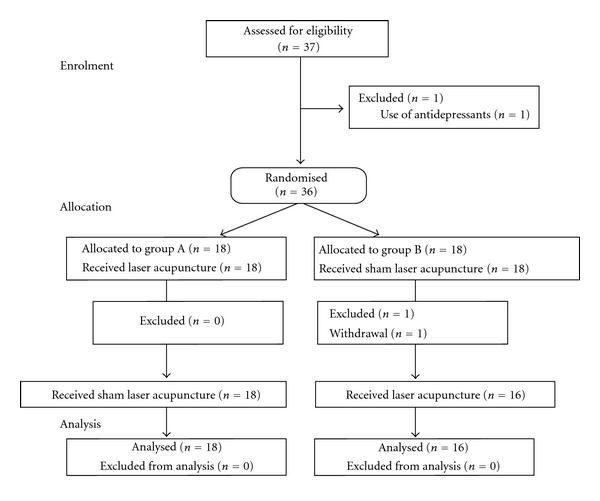
Enrolment in the study.

Thirty-four healthy volunteer subjects were recruited through advertisement in student dormitories and hospital staff social rooms. Subjects were randomly allocated into two groups. Volunteers received verum laser and sham laser treatment at three acupuncture points in a cross-over design. The interval between treatments was at least 3, and at most 4, days (Figure 1). The daily time points of measurement were kept equal. Subjects were informed that during one of the treatment episodes a sham laser would be used. 

In addition, we reviewed three of our randomized controlled trials for credibility (according to Vincent and Lewith [14]) between sham laser and needle acupuncture treatments.

#### 2.1.1. Randomized Allocation and Blinding

Volunteers were randomly allocated to start with either verum or sham laser treatment. The randomization list was compiled by an external physician and was not divulged to study practitioners or patients. The same physician prepared a series of sealed, sequentially numbered envelopes containing the treatment assignments. When a patient fulfilled the inclusion criteria the study physician opened the lowest numbered envelope to reveal the patient's group allocation. Blinding was achieved by neither the study practitioner nor the subject knowing if verum or sham laser acupuncture treatment was being used first.

#### 2.1.2. Subjects

Eligible volunteers had to be >18 years of age. Volunteers were excluded from the study if one or more of the following criteria were fulfilled: local and general contraindications for treatment such as participation in other studies, (laser-) acupuncture treatment in the week prior to the examination, any medication uptake (except contraceptives), pregnancy, coagulopathies, psychosis or other severe diseases. Exclusion criteria were assessed twice, orally by the examiner and in written form by the volunteers. Long hairs at the treatment spots were cut carefully with scissors without affecting the skin before treatment in order to avoid tactile stimulus during treatments.

#### 2.1.3. Ethics

The study protocol was approved by the Ethics Committee of the University of Munich, Germany. Written informed consent was obtained from all volunteers in accordance with the Declaration of Helsinki.

#### 2.1.4. Intervention

Interventions took place at the treatment rooms of the Interdisciplinary Pain Center, Department for Anaesthesia, University of Munich, Germany where a mean room temperature of 22°C is maintained.

**Verum Laser Acupuncture**

Laser acupuncture was performed with an infrared (IR) laser of low intensity (Seirin, 3B Scientific GmbH, Hamburg, Germany). The output power was 22-23 mW with a wavelength of laser light of 830 nm (continuous wave). The radiated area on the skin was 0.78 mm^2^ with an energy density of 113.0 kJ cm^−2^. Each treatment episode lasted 45 s, and radiation dosage at each point reached 1 kJ. This kind of laser device is also equipped by the manufacturer by default with a visual [red light, light-emitting diode (LED)] and acoustic signal. Treatment was performed in sequence (see below) on the right side at three commonly used acupuncture points: LI4, LU7 and LR3 [24]. The distance between skin and laser was 5 mm. Acupuncture points were localized without tactile irritation.

**Sham Laser Acupuncture**

Laser irradiation was deactivated by the manufacturer (Seirin, 3B Scientific GmbH, Hamburg, Germany). The visual (red light: ~587 nm) and acoustic functions were maintained in order to make this a sham procedure. The output power of the LED light was 20 *μ*W according to the manufacturer. Sham laser treatment was performed according to the verum treatment protocol.

#### 2.1.5. Outcome Measures

The main outcome measure was the ratio between correctly and incorrectly assigned treatments by subjects. In addition, after each point was treated, subjects had to comment if they noted any skin sensation during or immediately after the treatment and (if applicable) to quantify the intensity of any perceived sensation (i.e., deqi response) on a 10-fold visual analog scale (VAS; with 0 being no sensation and 10 being the strongest sensible sensation). At the end of each treatment session the volunteers were asked if they thought the laser device used was active or inactive.

We also analyzed three former publications for the credibility of sham laser and acupuncture treatments. We pooled the results of the four items comprising the credibility assessment according to Vincent.

#### 2.1.6. Statistical Analysis

Statistical analysis was done with SPSS statistical software system (SPSS Inc., Chicago, IL; version 15.0). Descriptive data are expressed as mean  ±  standard deviation.

Analysis of scaled data (e.g., perception of laser) was performed with the chi-square-Pearson test using Cohen's kappa to confirm *post hoc* the consistency of ratings. Continuous data (e.g., VAS) were analyzed with paired *t*-tests or the Wilcoxon-rank-sum test. Correlations were confirmed using the Spearman coefficient. Two-sided *P* < .05 were considered statistically significant.

Credibility assessment of former publications was rated according to their standardized mean difference with 95% confidence interval (CI) between needle acupuncture and sham laser acupuncture prior to and following treatment.

## 3. Results

### 3.1. Ratio between Correctly and Incorrectly Assigned Treatments

**Figure 2:**
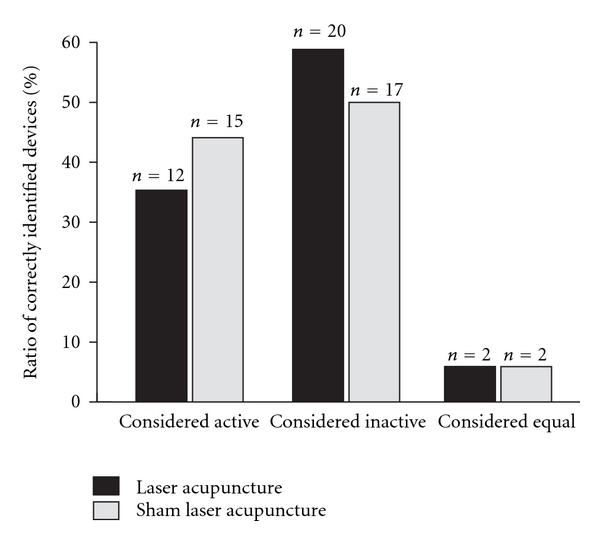
Ratio of correctly indentified devices (in percent) in the laser (black bars) and sham laser (grey) treatment group. Devices could be considered active, inactive or indistinguishable.

After the first treatment, 15 out of 34 subjects (44%) correctly identified the laser device as active, with 19 (56%) being incorrect (chi-square-Pearson *P* = .49). After the second treatment 14 out of 34 subjects (41%) correctly identified the laser device as active, with 16 (47%) being incorrect (*P* = .72). Four subjects (12%) felt that the two treatments were equal (Table 1). When comparing the ratio of correctly identified devices, laser acupuncture was identified correctly in 12 (35.3%) of all treatments, sham laser acupuncture was identified in 17 (50.0%) of all applications (Figure 2). Sham laser acupuncture was more likely to be supposed to be active (44.1%) than verum laser (35.3%; Figure 2). 

#### 3.1.1. Intensity of Perceived Sensations

Subjects receiving laser acupuncture perceived sensations (deqi) in 47 out of 102 treatments (46%) with a mean intensity of 2.34  ±  2.34 cm on the VAS. Sham laser caused sensations in 50 out of 102 cases (49%) with a mean intensity of 2.49  ±  2.36 cm on the VAS (Table 2). There was no significant difference demonstrated between these groups. 

#### 3.1.2. Comparison of Acupuncture-Experienced and -Naïve Subjects

Seventeen subjects had received needle acupuncture treatment in the past. They reported stronger sensations (3.2  ±  2.7 cm VAS) when compared to acupuncture-naïve subjects (*n* = 17; 1.4  ±  1.4 cm VAS, *P* = .0002). Experienced subjects were not able to identify the active treatment (29.4%) more frequently than naïve volunteers (41.2%, *P* = .32).

#### 3.1.3. Credibility Assessment of Sham Laser Acupuncture

**Figure 3:**
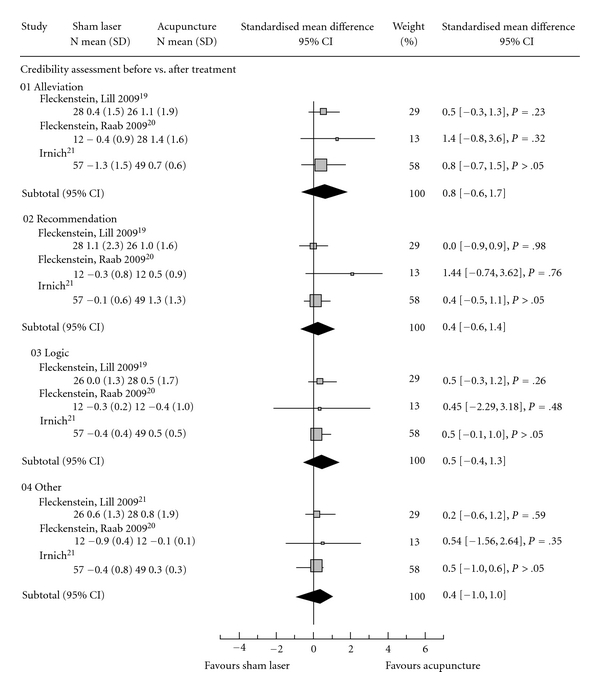
Indicating the standardized mean difference in credibility according to Vincent which is comprised of four items: (i) (Alleviation) How confident do you feel that this treatment can alleviate your complaint? (ii) (Recommendation) How confident would you be in recommending this treatment to a friend who suffered from similar complaints? (iii) (Logic) How logical does this treatment seem to you? (iv) (Other) How successful do you think this treatment would be in alleviating other complaints? SD, standard deviation; CI, 95% confidence interval.

A total of 186 patients assessed for credibility in three previous trials using sham laser acupuncture as control for needle acupuncture treatments were pooled in this analysis. When evaluating the credibility of sham laser, no significance in favor of one treatment could be shown (Figure 3; [10–12]). 

## 4. Discussion

Our study shows that healthy subjects were not able to reliably distinguish between laser and sham laser acupuncture treatment. Occurrence and intensity of perceived sensations at different acupuncture points was similar for both treatments. The credibility of sham laser acupuncture was equal to needle acupuncture. On the basis of these results we think that sham laser acupuncture can therefore serve as a valid control procedure for evaluating specific laser acupuncture effects.

Sham laser acupuncture treatment affords all requirements to produce the same non-specific effects as laser acupuncture treatment. As there were no differences between acupuncture-experienced and -naïve subjects in the ratio of identifying the true device from the sham procedure, future studies will not have to distinguish between different types of volunteers. The previous problem of blinding the examiner in hands-on treatment [14, 31] may have been solved by the study we present, since the treating physician was not able to identify the active laser. This means that the use of sham laser allows for a double-blinded treatment setting.

**Figure 4:**
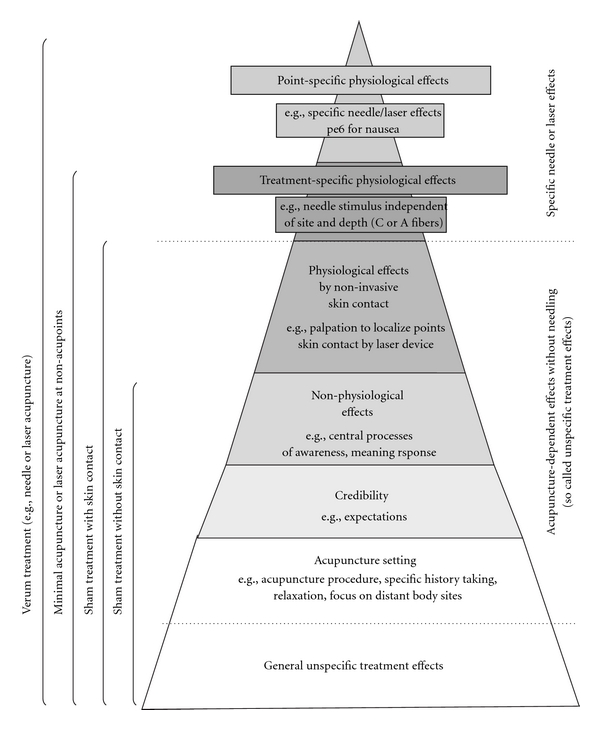
Specific and non-specific effects of needle acupuncture, laser- and sham laser acupuncture.

A major point for the validation of a placebo procedure is to prove that the credibility of expected treatment effects does not differ from the verum procedure. As our subjects were not able to distinguish between sham and verum procedures, it can therefore be reliably presumed that their credibility is equal. In addition, to relate the credibility of sham laser acupuncture to needle acupuncture we showed that the credibility of sham laser acupuncture was equal to that of acupuncture (Figure 4; [10–12]). The generally observed high values indicate that laser acupuncture may provoke strong “non-specific” effects because they have been observed in some needle acupuncture trials. This might also have an impact on the use of sham laser acupuncture as possible control in needle acupuncture trials. 

A characteristic of laser acupuncture treatment is the existence of so called deqi sensations during treatment. Both of our groups reported sensations in accordance with reported descriptions of classical deqi sensations [25, 26]. Deqi during needle acupuncture is reported in 57–71% of volunteers [25–27], which is comparable to the overall occurrence we observed in this trial (48%). The reason why the subjects treated with sham laser indicated deqi sensations remains unclear. It is our consideration that deqi sensations may be caused by central processes of awareness rather than the red light itself provoking deqi sensations directly within the skin.

Another importantly controlled factor in our trial was that manipulation while touching or even palpating the acupuncture points was strictly avoided in both groups. This is in contrast to another study that used sham laser as a control [10].

Different methods have been used to localize acupuncture points in clinical trials. Most of them include skin contact, which may confoundingly activate sensory receptors. This is especially important since it has been shown that slight touch may activate C-tactile afferents, which Olausson et al. supposed would produce a faint sensation of pleasant touch and consequently emotional, hormonal and affiliative responses [28]. Because of the structure of our trial, these influences can now be disregarded.

One limitation in interpreting our results could arise from our use of red light to strengthen the credibility of both sham and verum laser. The rationale behind this choice was the observation that the majority of infrared laser devices uses a red light in addition to the invisible laser emission, with the addition of visible red light serving as a valid indicator that the laser device is active. It could potentially be argued that this red light has physiological effects and may therefore not serve correctly as a sham treatment. The trial by Stelian et al. [29] lends support to this argument since they demonstrated red light as an active treatment in pain due to osteoarthritis when compared to invisible infrared and placebo light emitters. However, in this trial, treatment was applied 15 min twice a day for 10 days whereas a bulb emitting coherent narrow-band light was used in their study. In contrast, our sham device emitted incoherent light (LED) with an output power of 20 *μ*W and the treatment lasted 45 s. Due to this important difference regarding the intensity of red light application and the lack of conclusive basic research on the effect of red light applied to the skin for a short time, we think that our control procedure fits the definition of a sham. Nevertheless, further research would be appropriate to validate this.

Based on our results, we propose that sham laser acupuncture may serve as a credible sham procedure for needle acupuncture trials in which the aim is to evaluate needling effects *per se*. The treatment setting is equal to acupuncture in many respects, for example, individually history taking for choosing acupuncture points, attention, relaxation and concentration on body sites distant from the affected painful area. All these are potentially important factors for both physicians and volunteers [30].

There has also been controversial discussion on the subject of control procedures for clinical acupuncture trials [14, 27, 31]. The use of superficial or deep needle insertions at locations distant from real acupuncture points, known as minimal or sham acupuncture control, is not suitable as an inert placebo. Diverse physiological effects of needling stimulation have been observed off-site. This procedure is more likely to evaluate needling effects regarding the depth or the site of needle insertion. There are various physiological antinociceptive effects which are inevitable when using needle insertion or related techniques. Development of so-called “placebo needles” was an essential step in the methodology of research on mechanisms of acupuncture [27]. However, applying placebo needles in clinical trials creates methodological problems, for example, artificial setting, loss of credibility in acupuncture experienced by volunteers while still activating mechano-sensitive A*β*-fibers or C-tactile afferents, which by itself can activate pain-inhibitory systems [32, 33]. In addition, all these methods share the impossibility of successfully blinding the therapists. The use of sham laser, in contrast, avoids activation of such non-specific physiological effects, and double-blinding of the therapist and patient is easily achieved.

Nonetheless, sham laser acupuncture has been shown to have clinically relevant effects in our formerly published trials [10–12]. In one large trial comparing needle acupuncture to sham laser and massage, 32% of patients treated with sham laser experienced a decrease of motion-related pain of at least 50% compared to baseline [12]. In the primary analyses, needle acupuncture was also not superior to sham laser acupuncture. Vickers reanalyzed this study using linear regression models and demonstrated that acupuncture needling was of clear benefit, attributing a measurable placebo effect to the sham laser treatment [34]. In the two other trials for sham laser as placebo [10, 11], we also found considerable placebo effects.

As sham laser has no sensory input on the peripheral nervous system, its effects, which have been observed both in our trial as well as in the trials of other groups, can only be explained by non-physiological effects. We could call them psychological, non-specific or setting effects, and surely they are diverse. With ongoing research, it is becoming clearer that these effects are valid and context sensitive. What is more, it appears that such effects can be distinguished as whether produced by sham laser or physiological needling. The effects of site (e.g., at classical acupuncture points), depth (deep versus superficial versus slight touch) or different stimulation techniques remain the subject of trials comparing different needling techniques (e.g., verum, sham, minimal).

However, the use of sham laser as a control for acupuncture trials may raise a further limitation. Technical devices are not directly comparable to the manual skill of acupuncture, and they are also liable to be held in different regard by volunteers when concerning their relative treatment effects [35]. The only argument against this that we can provide is that credibility of sham laser is comparable to that of needle acupuncture.

Overall, we do recognize that sham laser is a not a perfect placebo for acupuncture trials; however, it offers so many reasonable advantages that it deserves to be strongly considered as a standard means of control.

## 5. Conclusion

Sham laser acupuncture can serve as a valid placebo control in laser acupuncture studies. Due to their similar credibility and the lack of sensory input on the peripheral nervous system, we also believe that sham laser acupuncture can serve as a sham control for acupuncture trials when there is a need to evaluate the effects of needling *per se*.

## Figures and Tables

**Table 1:** Amount (*n*) of identified treatments (in %).

	After first treatment	After second treatment
Identified correctly	15 (44.1)	14 (41.2)
Identified incorrectly	19 (55.9)	16 (47.1)
Identified as equal	0 (0.0)	4 (11.8)

**Table 2:** Overall occurrence (in %) and intensity (mean  ±  SD) of sensations, distribution to specific acupuncture points (*n*).

	Laser acupuncture	Sham laser acupuncture
Occurrence	46.1	49.0
Intensity	2.3 ± 2.3	2.5 ± 2.4
Distribution		
LI4	14	11
LU7	18	24
LR3	15	15
